# Silymarin Protects Mouse Liver and Kidney from Thioacetamide Induced Toxicity by Scavenging Reactive Oxygen Species and Activating PI3K-Akt Pathway

**DOI:** 10.3389/fphar.2016.00481

**Published:** 2016-12-15

**Authors:** Shatadal Ghosh, Abhijit Sarkar, Sudip Bhattacharyya, Parames C. Sil

**Affiliations:** Division of Molecular Medicine, Bose InstituteKolkata, India

**Keywords:** silymarin, thioacetamide, oxidative damage, liver, PI3k/Akt, kidney, MAPKs, apoptosis

## Abstract

Silymarin (SMN) has been shown to possess a wide range of biological and pharmacological effects. Besides, SMN has antioxidant and free radical scavenging activities. Thioacetamide (TAA) is a well-documented liver toxin that requires oxidative bioactivation to elicit its hepatotoxic effect which ultimately modifies amine-lipids and proteins. Our study has been designed in a TAA exposed mouse model to investigate whether SMN could protect TAA-induced oxidative stress mediated hepatic and renal damage. Results suggest that TAA generated reactive oxygen species (ROS), caused oxidative stress and induced apoptosis in the liver and kidney cells via JNK as well as PKC and MAPKs signaling. All these detrimental effects of TAA could, however, be suppressed by SMN which not only scavenged ROS but also induced PI3K-Akt cell survival pathway in the liver and prevented apoptotic pathways in both the organs. Histological studies, collagen staining and DNA fragmentation analysis also supported our results. Combining, we say that SMN possess beneficial role against TAA mediated hepatic and renal pathophysiology.

## Introduction

Silymarin (SMN), an extract of *Silybum marianum*, has well-known hepatoprotective properties (Letteron et al., 1990; Muriel and Mourelle, 1990a). Being a standardized mixture of flavonolignans, including silibinin, silydianin, sosilibinin, and silychristin, SMN shows free radical scavenging property and cell membrane stabilizing activity (Muriel and Mourelle, 1990a,b; Abdel-Moneim et al., 2015; Reina and Martinez, 2016). At the molecular level, it is believed that SMN stimulates RNA and protein synthesis leading to faster regeneration of damaged liver tissue. SMN also modulates TNF-α associated inflammation pathway. However, the vivid molecular pathways through which SMN exerts its effects are still not clear. The present study has been aimed to investigate the effects of SMN during thioacetamide (TAA) induced hepatotoxicity in details. TAA is used as a fungicide and also serves as a source of sulfur in industries and pharmaceuticals (Kadir et al., 2011). Most importantly, it is a typical hepatotoxin that dose dependently causes centrilobular cell death accompanied by enhanced plasma transaminases and bilirubin. It has been observed that acute exposure with TAA causes necrosis while chronic exposure causes apoptosis in the liver (Moreira et al., 1995). To elicit these effects, TAA requires oxidative bioactivation into its *S*-oxide (TASO) ultimately leading to its chemically reactive *S,S*-dioxide (TASO2) form (Hajovsky et al., 2012). These metabolites are then distributed among several organs including plasma, liver, kidney, adrenals, bone marrow, and other tissues (Barker and Smuckler, 1974) and modify amine-lipids along with proteins leading to further systemic oxidative stress.

Liver plays a crucial role in the metabolic elimination of most of the currently used drugs and many other foreign compounds, thereby making it one of the most viable target organs for toxicity. Therefore, hepatoprotective agents are necessary in clinical therapy to fight against increasing liver toxic injury (Sinha et al., 2007b). Moreover, vast knowledge of the underlying mechanism of hepatotoxicity has to be attained. Unfortunately, necessary studies, in this field, are limited because of the lack of satisfactory experimental models. Nevertheless, some chemical toxins (like carbon tetrachloride, acetaminophen, and TAA) are often used to create experimental hepatocyte injury models in both *in vivo* and *in vitro* conditions (Ledda-Columbano et al., 1991; Kucera et al., 2006; Domenicali et al., 2009; Rousar et al., 2009). However, detail mechanism was not investigated thoroughly. Therefore, in the present study, we have taken a detailed mechanistic approach to explore the molecular signaling pathways as well as histopathological examinations to find out how SMN exerts its beneficial effects. It is to be mentioned that, we have also observed renal dysfunctions with the exposure of TAA and investigated this renal toxicity too in details. For this purpose, the protective action of SMN was evaluated on the basis of several parameters like the activity of antioxidant enzymes and cellular antioxidant power (FRAP); increase of the body weight and cellular non-enzymatic antioxidant (GSH) content; amelioration of the tissue damage (histological assessment) and most importantly the interaction of different signaling molecules associated with the ameliorative role of SMN.

## Materials and Methods

### Materials

#### Chemicals

Silymarin, TAA, BSA, Bradford reagent, anti-Bcl-2, anti- Bcl-xL, anti-Bad, and anti-Bax antibodies were purchased from Abcam (UK). Other antibodies were purchased from Sigma-Aldrich Chemical Company (St. Louis, MO, USA). Kits for measurement of blood glucose and LDH were purchased from Span Diagnostic Ltd., India. All other chemicals were bought from Sisco Research Laboratory, India.

#### Animals

Adequate numbers of adult male Swiss Albino mice weighing ~20–25 g were acclimatized under laboratory conditions for 2 weeks before any experiment. Animals were maintained under standard conditions of temperature (23 ± 2°C) and humidity (50 ± 10%) with alternating 12 h light/dark cycle. The animals were given free access to water and fed standard pellet diet (Agro Corporation Private Ltd., Bangalore, India). All the experiments that had been conducted with animals followed the guidelines approved by the IAEC (Institutional Animal Ethical Committee), Bose Institute, Kolkata [IAEC/BI/3(I) cert./2010] and the study had been approved by both CPCSEA (Committee for the Purpose of Control & Supervision on Experiments on Animals) Ministry of Environment and Forests, New Delhi, India (1796/PO/Ere/S/14/CPCSEA) and IAEC.

### Methods

#### Experimental Design for *in vivo* Treatments

To design the *in vivo* experimental study the mice were randomly assigned to four groups and treated as follows:

Group 1: Normal group: mice received neither TAA nor SMN, received vehicle only.
Group 2: Silymarin group: mice received only SMN (150 mg/kg body weight in olive oil) orally for 8 weeks (simultaneously with Group 4).
Group 3: Thioacetamide group: mice received TAA injection at a dose of 100 mg/Kg body weight twice a week for 56 days (Ansil et al., 2011).
Group 4: TAA and SMN group (post-treatment group): mice received SMN (150 mg/kg body weight in olive oil) orally for 8 weeks after TAA administration twice a week for 56 days.

After the experimental periods animals were sacrificed by cervical dislocation and livers and kidneys were collected.

#### Determination of Dose and Time-Dependent Activity of SMN by ALP Assay

**FIGURE 1:**
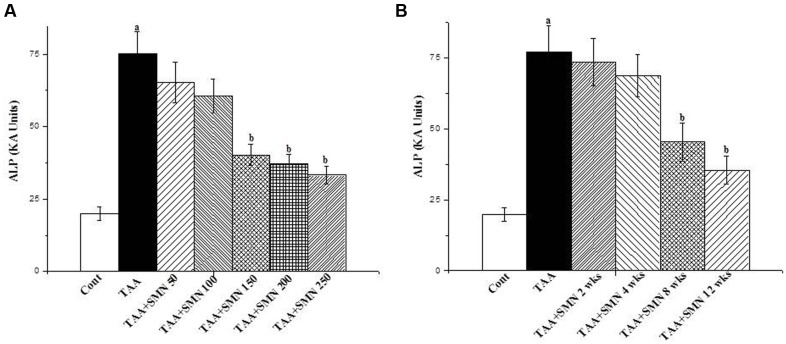
**(A)** Representation of the dose dependent study of SMN on ALP level in TAA-treated pathophysiology in the serum of the experimental mice. Cont: measurement of serum ALP in normal mice, TAA: measurement of serum ALP in TAA administered mice, TAA+SMN 50, TAA+SMN 100, TAA+SMN 150, TAA+SMN 200, and TAA+SMN 250: measurement of serum ALP in mice which are treated with SMN at a dose of 50, 100, 150, 200, and 250 mg/kg body weight, orally, respectively, after TAA administration at a dose of 100 mg/kg body weight, i.p. **(B)** Representation of the time dependent study of SMN on ALP activity in TAA-exposed pathophysiology in the serum of the experimental mice. Cont: measurement of serum ALP in normal mice, TAA: measurement of serum ALP in TAA exposed mice, TAA+SMN 2 week, TAA+SMN 4 week, TAA+SMN 8 week, and TAA+SMN 12 week: measurement of serum ALP in mice which are treated with SMN at a dose of 150 mg/kg body weight, orally for 2, 4, 8, 12, and 16 weeks, respectively. “a” indicates the significant difference between the normal control and TAA exposed groups, “b” indicates the significant difference between TAA exposed (toxin control) and SMN post-treated groups. Each column represents mean ± SEM, *n* = 6; (*p*^a^ < 0.05, *p*^b^ < 0.05).

For this study, mice were randomly distributed into seven groups each consisting of six animals. The first two groups served as normal control (receiving vehicle only) and toxin control (receiving TAA at a dose of 100 mg/kg body weight thrice a week in citrate buffer, pH 4.5, i.p.), respectively. The remaining five groups of animals were treated with five different doses of SMN (50, 100, 150, 200, and 250 mg/kg body weight) for 56 days after 3 weeks followed by TAA administration (**Figure 1A**).

For the time-dependent study, mice were randomly distributed into six groups each consisting of six animals. The first group served as normal control receiving only vehicle and the second group served as toxin control exposed to TAA at a single dose of 100 mg/kg body weight thrice a week in citrate buffer, pH 4.5, i.p. The remaining four groups of animals were treated with SMN orally at a dose of 150 mg/kg body weight, once daily for 2, 4, 8, and 12 weeks after the last TAA administration (**Figure 1B**).

#### Measurement of Body Weight

The body weights of all the experimental animals from each group were measured.

#### Preparation of Liver Tissue Homogenate

Liver tissues from experimental animals were first homogenized (1:4, w/v) in ice-cold 0.1 M phosphate buffer (pH 7.4) containing 2 mM EDTA. The homogenate was later centrifuged at 10,000 × *g* for 30 min at 4°C. The supernatant was collected and recentrifuged at 105,000 × *g* for 55 min. The subsequent pellets containing microsomal fractions were suspended in 0.25 mM sucrose solution containing 1 mM EDTA and stored at -80°C until further use. The resulting supernatant containing the cytosolic fraction was also collected and used for the experiments. The protein contents of both the cytosolic and microsomal fractions were estimated by the method of Bradford (1976) using crystalline BSA as standard.

#### Determination of Hepatic and Renal Markers and Nitric Oxide Production

Specific markers associated with hepatic and renal dysfunction, e.g., AST, ALT, ALP activities, BUN, creatinine, and albumin were estimated by using standard kits. LDH activity was estimated according to the method of Kornberg (1955). The hepatic nitric oxide (NO) level was indirectly assessed using the Griess reaction (Green et al., 1982; Suresh and Das, 2006).

#### Estimation of Lipid Peroxidation

Lipid peroxidation in terms of malondialdehyde (MDA) formation was estimated following the method of Esterbauer and Cheeseman (1990).

#### Assay of Cellular Metabolites

GSH content was measured, according to the method of Ellman (1959).

GSSG content was estimated following the method of Hissin and Hilf (1976).

#### Determination of *in vivo* Antioxidant Power by FRAP Assay

Ferric Reducing Antioxidant Power assay was performed following the method as described by Benzie and Strain (1999).

#### Assay of Antioxidant Enzymes

Activities of different antioxidant enzymes (SOD, CAT, GST, GR, and GPx) have been assessed using the liver tissue homogenates. SOD activity was evaluated as described previously (Manna et al., 2013). One unit of SOD activity could be defined as the enzyme concentration that required to attenuate chromogen production by 50% in 1 min under the assay conditions.

Catalase activity was estimated by following the decomposition of H_2_O_2_ at 240 nm for 10 min according to the method of Bonaventura et al. (1972). One unit of CAT activity is defined as the amount of enzyme, which reduces one μmol of H_2_O_2_ per minute.

Glutathione *S*-transferase activity was assayed based on the conjugation reaction with GSH in the primary step of mercapturic acid synthesis (Habig et al., 1974). GST activity was expressed as μmoles of CDNB conjugate formed/min/mg protein.

Glutathione reductase activity was determined following the method of Smith et al. (1988).

Glutathione peroxidase activity was measured according to the method of Flohé and Günzler (1984) using H_2_O_2_ and NADPH as substrates.

#### Determination of Radical-Scavenger Activity of SMN in a Cell-Free System

##### Superoxide radical-scavenging activity

The superoxide radical-scavenging activity of SMN was measured according to the method of Siddhuraju and Becker (2003) and Ghosh et al. (2010). Briefly, the reaction mixture contained 0.1 M phosphate buffer, pH 7.4, 50 μM nitro blue tetrazolium, 468 μM NADH, 60 μM phenazine methosulfate, and different concentrations of SMN. The mixture was then incubated in the dark for 10 min at room temperature and the absorbance was read at 560 nm. Results were expressed as percentage inhibition. All experiments were performed in triplicate.

##### Hydroxyl radical-scavenging activity

The hydroxyl radical-scavenging activity of SMN was measured according to the method of Nash (1953). Briefly, hydroxyl radicals content was measured by the amount of formaldehyde (detected spectrophotometrically at 412 nm) produced from the oxidation of dimethyl sulfoxide (DMSO). All experiments were performed in triplicate.

##### Nitric oxide radical-scavenging activity

The nitric oxide radical-scavenging activity of SMN was determined according to the method of Green et al. (1982). The amount of the chromophore formed was detected spectrophotometrically at 540 nm. Results were expressed as percentage. All experiments were performed in triplicates.

#### DNA Fragmentation Assay

DNA fragmentation assay was performed following the method of Das and Sil (2012).

#### Determination of Mitochondrial Membrane Potential (_Δψ_m__)

Mitochondria were isolated following the method of Hodârnâu et al. (1973). In short, the membrane potential (_Δψ_m__) was measured using a FACS machine with an argon laser excitation at 488 and 525 nm band pass filter. The evaluation of the mitochondrial membrane potential (_Δψ_m__) was determined on the basis of cell preservation of the fluorescent probe JC-1.

#### Immunoblotting

Proteins (50 μg) from each sample were separated by 10% SDS-PAGE and transferred into PVDF membranes. Membranes were then blocked using BSA and incubated separately with primary antibodies of anti caspase-3, anti- PARP, and anti-Bcl-xL (1:1000 dilution), anti cytochrome *c* (1:1000 dilution), anti Bad (1:1000 dilution), anti Bax (1:1000 dilution), anti Bcl-2 (1:1000 dilution), etc., at 4°C for overnight. All the primary antibodies were detected against HRP-conjugated secondary antibody using the HRP substrate ECL solution (Sarkar et al., 2016).

#### Measurement of Hepatic Collagen Content

Hepatic collagen content was estimated following the method of Gascon-Barré et al. (1989). Briefly, liver sections (15 μm thick) were taken from liver blocks embedded in paraffin and then deparaffinised producing tissue areas of ~50–100 mm^2^. These were then incubated in 0.04% (w/v) solution of fast green in saturated picric acid for about 15 min. After that, all the sections were washed with distilled water, incubated with fast green FCF 0.1% (w/v) and sirius red F3B 0.04% (w/v) for about 30 min, then washed again with distilled water until the eluted fluid becomes completely colorless. Then 1 ml of 0.05 N NaOH in 50% (v/v) aqueous methanol was added in each tube and mixed gently. The eluted color was quantified by the help of a spectrophotometer at two wavelengths *viz*. 530 and 605 nm (corresponding to the absorbance maxima of sirius red and fast green, respectively).

#### Isolation of Primary Mouse Hepatocytes

Hepatocytes were isolated from mice liver by the perfusion technique with collagenase type I at 37°C (Sinha et al., 2007a). Only the preparations with 90% or more viable cells were used for subsequent experiments. Cells were seeded onto culture plates precoated with collagen. Cell density was 2 × 10^5^ cells/well for 24 well plates and 1 × 10^6^ cells/well for six well plates. The cells were cultured and maintained in William’s medium E, supplemented with 0.3 μM insulin, 10% FBS, 0.1, μM dexamethasone and 1% streptomycin/penicillin at 37°C, 90% humidity and 5% CO_2_. All experiments were performed 24 h after cell attachment allowing the formation of a monolayer.

#### Assessment of Hepatocyte Viability

Viability of hepatocytes was determined by the MTT assay. Briefly, 250 μl of MTT solution (300 mg/ml) was added to the culture medium (200 μl in each well) 1 h before the end of 12 h treatment and incubated at 37°C for 30 min. After that, supernatants were discarded and 200 μl of DMSO was added and mixed thoroughly to dissolve the crystals. Then, absorbance was taken at 570 and 630 nm.

#### RNA Extraction and Reverse Transcriptase PCR (RT-PCR)

RNA was extracted from the liver and kidney tissue of all four groups of mice using the TRIzol reagent, following the manufacturer’s protocol (Invitrogen, Carlsbad, CA, USA). The amount of RNA was estimated spectrophotometrically using nanodrop, HellmaTrayCell Type 105.810 (Hellma Analytical). Two microgram of extracted RNA from each sample was converted to cDNA using Thermo Scientific Verso cDNA synthesis kit (Thermo Scientific, USA). Thermal cycling was performed following the sequences: 95°C for 5 min (initial denaturation) followed by the 35 set of cycles: 95°C for 30 s (denaturation), 55°C for 30 s (primer annealing), and 72°C for 45 s (primer extension). After the cycles the time for DNA extension was given 5 min at 72°C. After the extension, PCR amplification products were then held at 4°C and subjected to electrophoresis on 1.5% agarose gel.

#### Histological Studies

Portions of livers and kidneys were fixed in 10% v/v neutral buffered formalin solutions for 24 h. Standard techniques were followed for processing the tissue and preparation of paraffin blocks. Fibrosis and necroinflammation were evaluated in sections (5 μm thick) stained with Masson’s trichrome and hematoxylin–eosin, respectively, using the Ishak-modified HAI system (Mannan et al., 2014). Mean values were calculated from each of six glomeruli per section.

#### Statistical Analysis

All experimental values have been represented as mean ± S.E.M (*n* = 6). Data on biochemical investigation were analyzed using analysis of variance (ANOVA) and the group means were compared by Tukey Test. *P*-values of 0.05 or less were considered significant.

## Results And Discussion

A number of naturally occurring agents have been studied to assess their hepato-protective roles. In many cases TAA has been used to create a model of hepato-toxicity. These agents include reynosin (Lim et al., 2013), Ginkgo biloba leaves extract (Al-Attar, 2012) and ethanolic and aqueous extracts of *Phyllanthus acidus* (L.) Skeels (*P. Acidus*; Jain and Singhai, 2011), etc. An imidazolium salt, 1,3-diisopropylimidazolium tetrafluoroborate (DPIM) has also been shown to reduce TAA-induced mouse liver injury (Ding and Zhuo, 2013). Apart from these agents, SMN itself has been shown to possess a remarkable protective effect toward liver as well as other organs. But how SMN exerts its effects are not fully clear especially in case of cellular signaling processes.

In the present study, we investigated whether the acute hepatotoxic and associated nephrotoxic effect of TAA could be prevented or cured by SMN and if yes what molecular pathways SMN uses.

### Body Weight and Cellular Leakage

**FIGURE 2:**
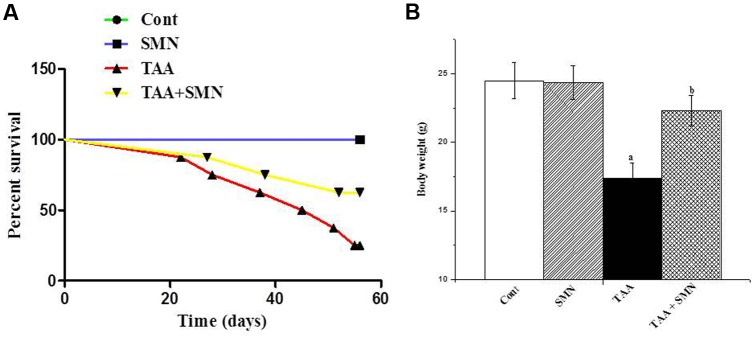
**(A)** Survival curve of the animals obtained from the data of the 8 weeks study. **(B)** Effect of silymarin treatment on the body weight of animals of different groups.

**Figure 2A** shows how SMN increases the survival percentage of TAA exposed animals. **Figure 2B** shows that TAA exposure significantly reduced the body weight of the experimental animals. SMN, however, could protect mice from this TAA induced weight loss.

**Table 1** demonstrates significant elevation of serum ALP, ALT, AST, and LDH activities in the livers and BUN and creatinine levels in the kidneys (**Table 2**) of the animals exposed to TAA that altered the transport function and membrane permeability of respective organs. Leakage of these enzymes was elevated significantly compared to normal animals upon TAA exposure indicating severe tissue damage. However, the activities of these markers in SMN treated group remained almost the same as normal values. Albumin level was also decreased in TAA exposed group. SMN post-treatment decreased these alleviated values of the hepatic and renal markers toward the normal value.

**Table 1:** Effect of TAA and SMN on liver marker enzymes.

Parameters	Cont	SMN	TAA	TAA+SMN
ALP	14.45 ± 1.25	16.36 ± 1.31	39.12 ± 2.64^a^	18.35 ± 1.51^b^
ALT	58.67 ± 1.51	69.23 ± 1.67	140.04 ± 2.31^a^	81.75 ± 2.11^b^
AST	132.78 ± 4.15	145.65 ± 5.43	361.73 ± 7.75^a^	213.47 ± 7.91^b^
LDH	172.35 ± 5.35	194.63 ± 6.19	510.74 ± 9.68^a^	247.65 ± 7.27^b^
Albumin	3.42 ± 0.06	3.31 ± 0.07	2.12 ± 0.10^a^	2.97 ± 0.09^b^
NO production	28.05 ± 0.76	29.03 ± 0.81	71 ± 1.78^a^	38 ± 1.13^b^
FRAP	100 ± 8,91	100 ± 13.67	100 ± 67.37^a^	100 ± 17.25^b^

*^a^“a” values differs significantly from normal control (p^a^ < 0.05); ^b^“b” values differs significantly from toxin control (p^b^ < 0.05). Values are expressed as mean ± SEM, for six animals in each group.*

**Table 2:** Effect of TAA and SMN on renal markers.

Parameters	Cont	SMN	TAA	TAA+SMN
Creatinine (mg/dl)	0.56 ± 0.01	0.58 ± 0.01	1.79 ± 0.036^a^	0.79 ± 0.031^b^
BUN (mg/dl)	19.12 ± 0.35	20.13 ± 0.36	36.46 ± 0.69^a^	23.03 ± 0.39^b^

Nitric oxide level was also increased significantly in TAA exposed animals. This increased NO production due to the oxidative stress caused by TAA exposure, in turn, might induce cell death when NO interacts with superoxide anion to form peroxynitrite. However, SMN post-treatment effectively lowers NO production (**Table 1**).

Measurement of lipid peroxidation, a biomarker of oxidative stress, gives a quantitative estimation of ROS mediated cellular injury. SMN treatment significantly inhibited hepatic and renal lipid peroxidation compared to TAA exposed animals (**Tables 3** and **4**).

**Table 3:** Effect of TAA and SMN on antioxidant enzymes activities in liver tissue.

Parameters	Cont	SMN	TAA	TAA+SMN
SOD	164.59 ± 2.73	165.59 ± 3.51	115.25 ± 4.37^a^	144.60 ± 3.29^b^
CAT	154.54 ± 3.03	156.51 ± 3.02	87.45 ± 3.39^a^	129.14 ± 2.68^b^
GPx	84.93 ± 1.25	86.23 ± 1.88	48.61 ± 2.13^a^	75.73 ± 1.77^b^
GR	40.46 ± 0.74	40.15 ± 0.98	18.99 ± 1.52^a^	28.14 ± 1.41^b^
GST	3.05 ± 0.05	2.84 ± 0.06	1.37 ± 0.08^a^	2.56 ± 0.07^b^
MDA	2.59 ± 0.05	2.57 ± 0.06	5.34 ± 0.15^a^	3.13 ± 0.16^b^
Redox ratio	9.11 ± 0.37	8.98 ± 0.38	4.46 ± 0.23^a^	7.36 ± 0.26^b^

*^a^“a” values differs significantly from normal control (p^a^ < 0.05); ^b^“b” values differs significantly from toxin control (p^b^ < 0.05). Values are expressed as mean ± SEM, for six animals in each group.*

**Table 4:** Effect of TAA and SMN on antioxidant enzymes activities in kidney tissue.

Parameters	Cont	SMN	TAA	TAA+SMN
SOD	175.45 ± 5.69	170.37 ± 5.47	129.34 ± 6.71^a^	161.37 ± 7.36^b^
CAT	132..56 ± 4.61	133.61 ± 4.54	76.32 ± 4.03^a^	117.59 ± 5.71^b^
GPx	125.91 ± 6.13	122.03 ± 5.51	69.23 ± 4.81^a^	108.15 ± 5.13^b^
GR	115.35 ± 4.12	109.68 ± 4.05	67.36 ± 3.79^a^	98.26 ± 4.21^b^
GST	3.09 ± 0.06	2.98 ± 0.07	1.99 ± 0.08^a^	2.65 ± 0.09^b^
MDA	2.51 ± 0.1	2.67 ± 0.14	6.31 ± 0.41^a^	3.21 ± 0.47^b^
GSH	25.08 ± 1.07	26.13 ± 1.09	18.32 ± 0.87^a^	22.04 ± 0.97^b^
Redox ratio	32.23 ± 1.25	32.06 ± 1.31	21.13 ± 1.41^a^	28.38 ± 1.46^b^
FRAP	100 ± 4.53	98.65 ± 4.32	47.25 ± 5.13^a^	89.05 ± 9.33^b^

*^a^“a” values differs significantly from normal control (p^a^ < 0.05); ^b^“b” values differs significantly from toxin control (p^b^ < 0.05). Values are expressed as mean ± SEM, for six animals in each group.*

### Effect on Ferric Reducing Antioxidant Power (FRAP)

Ferric reducing antioxidant power assay gives an indication of the antioxidant capacity (Benzie and Strain, 1999). In the present study, we performed FRAP assay to evaluate the antioxidant capacities of the hepatic as well as renal tissues of different groups. Results showed TAA exposure caused a significant reduction in FRAP value. However, SMN treatment brought the FRAP value back toward normal (**Tables 1** and **4**).

### Effect on Cellular GSH and GSSG Levels

Glutathione, a cysteine-containing tri-peptide, is synthesized within the cells from its constituent amino acids and plays a crucial role in maintaining cellular antioxidant capacity. Both the hepatic and renal GSH level decreased in TAA exposed group resulting in the decrease in the GSH/GSSG ratio significantly compared to normal. SMN treatment, however, effectively elevated the level of GSH toward its normal value. In SMN group, the GSH level remained almost the same as normal (**Tables 3** and **4**).

### Effect on Cellular Antioxidant Enzymes

We have measured the cellular antioxidant enzymes (CAT, SOD, GPx, GR, and GST) believed to fight oxidative stress directly. Results showed the least antioxidant enzyme activities in the TAA group both in case of hepatic and renal tissues (**Tables 3** and **4**). SMN post-treatment restored those enzyme activities toward the normal value. In the SMN group those values were almost comparable to normal.

The results from the studies performed so far, indicate that TAA administration caused significant amount of oxidative stress in liver and also in kidney. Literature (Natarajan et al., 2006a,b; Amirtharaj et al., 2016) also supports our indication.

But how SMN ameliorates oxidative stress remained unclear. Some earlier reports (Abdel-Moneim et al., 2015) indicated that it could be due to its free radical scavenging capacity. So, we have investigated its different radical-scavenging (hydroxyl, superoxide, and nitric oxide radical) properties in a cell-free system.

The results showed the radical-scavenging properties of SMN itself as evidenced from its hydroxyl, superoxide and nitric oxide radical scavenging ability in a cell-free system.

**FIGURE 3:**
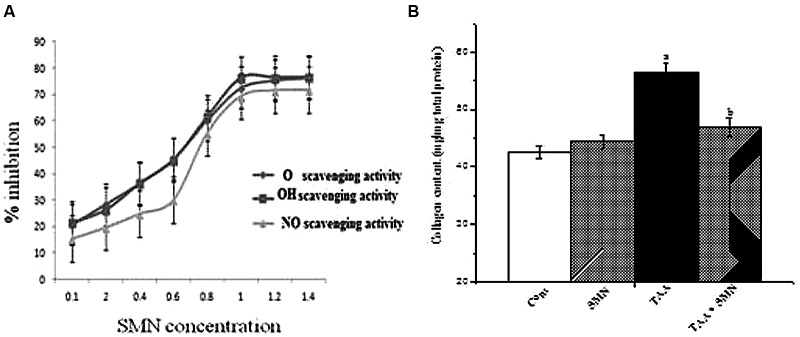
**(A)** Superoxide radical (O_2_^-^), hydroxyl radical (OH•), and nitric oxide (NO•) scavenging activity of SMN in the cell-free system. Each point represents the mean ± SEM, *n* = 6; **(B)** Collagen content of liver from various groups of animals.

In short, our experiments indicate that SMN treatment gives protection against the elevation in the level of ROS production as well as NO production in liver and kidney. However, the beneficial effects of SMN may arise partly from its radical scavenging action (**Figure 3A**).

### Collagen Content

The results of our study showed a significant increase in hepatic and renal collagen content as a result of TAA exposure twice weekly for 8 weeks. SMN treatment, however, decreased the collagen content in liver and kidney, though this effect was not significant in case of kidney (data not shown).

Together all of these results imply that SMN protected mouse liver and kidney against TAA induced pathophysiology mainly by exerting its antioxidant properties (**Figure 3B**).

### TAA-Induced Hepatocellular Apoptosis

**FIGURE 4:**
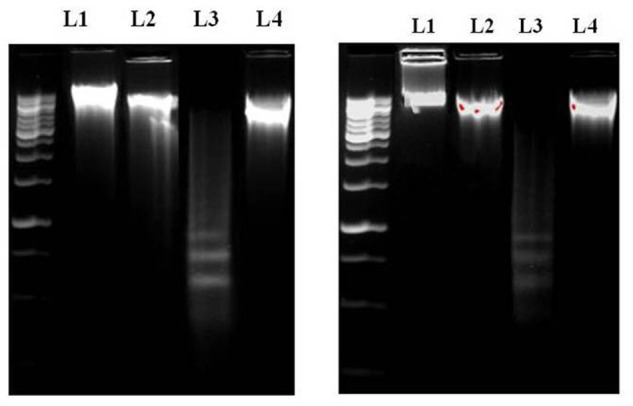
**(A)** DNA [isolated from liver **(A)** and kidney **(B)**] fragmentation pattern on agarose/EtBr gel. Lane 1 marker (1 kb DNA ladder), lanes 2, 3, 4, and 5 DNA isolated from normal, SMN treated, TAA administered, SMN post-treated mice, respectively. Arrows indicate ladder formation. SMN decreased all the TAA-induced pro-apoptotic events in hepatic and renal tissue.

Then, we wanted to know the mode of cell death. So, we have done DNA gel electrophoresis to investigate the nature of cell death (necrosis and/or apoptosis). Results showed that DNA isolated from TAA-exposed mice produced ladder (a hallmark of apoptosis) on the agarose gel. Results suggest the anti-apoptotic nature of SMN against TAA-induced hepatic and renal pathophysiology (**Figures 4A,B**).

### Histopathological Findings

**FIGURE 5:**
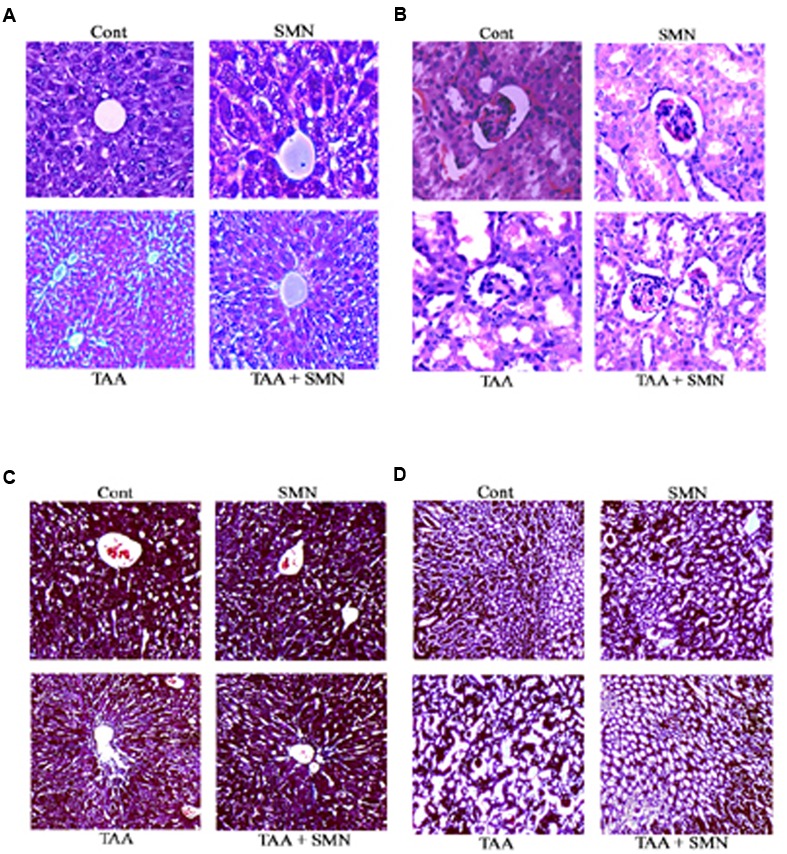
**Histological observations.** Hematoxylin and eosin staining of liver **(A)** and kidney **(B)**. Cont: normal tissues (10×); TAA: TAA exposed (100 mg/kg body weight for 56 days) tissues (10×); SMN: SMN treated (150 mg/kg body weight for 56 days) tissues (10×); TAA + SMN: tissues from animals treated with SMN (150 mg/kg body weight in olive oil orally for 8 weeks) after TAA administration twice a week for 56 days (10×). Masson’s Trichrome staining of liver **(C)** and kidney **(D)**. Cont: normal tissues (10×); TAA: TAA exposed (100 mg/kg body weight for 56 days) tissues (10×); SMN: SMN treated (150 mg/kg body weight for 56 days) tissues (10×); TAA + SMN: tissues from animals treated with SMN (150 mg/kg body weight in olive oil orally for 8 weeks) after TAA administration twice a week for 56 days (10×).

Histopathological studies of the sections from mouse liver exposed to TAA showed (in HE staining) severe tissue damage and vacuolar degeneration. SMN post-treatment attenuated the hepatic injury and showed significant protection of the hepatic cells from apoptotic death. There were no such alterations in SMN group compared to normal (**Figure 5A**).

Likewise, in case of kidney samples, TAA administration also showed impaired renal morphology including severe tubular epithelial cell death associated with inflammatory cell infiltration and glomerular congestion. SMN post-treatment effectively protected the kidney tissue from these alterations. Only SMN, however, did not show any significant change compared to normal (**Figure 5B**).

In Masson’s Trichrome stains, collagen was found to be deposited only in the liver blood vessels in the control and SMN groups. But TAA administration caused a marked increase in collagen accumulation primarily in the periportal regions. However, SMN post-treatment for 8 weeks ameliorated this collagen deposition both in the liver and kidney tissues (**Figures 5C,D**).

### TAA Stimulated Pro-apoptotic Signaling via the Activation of JNK: Protective Role of SMN

**FIGURE 6:**
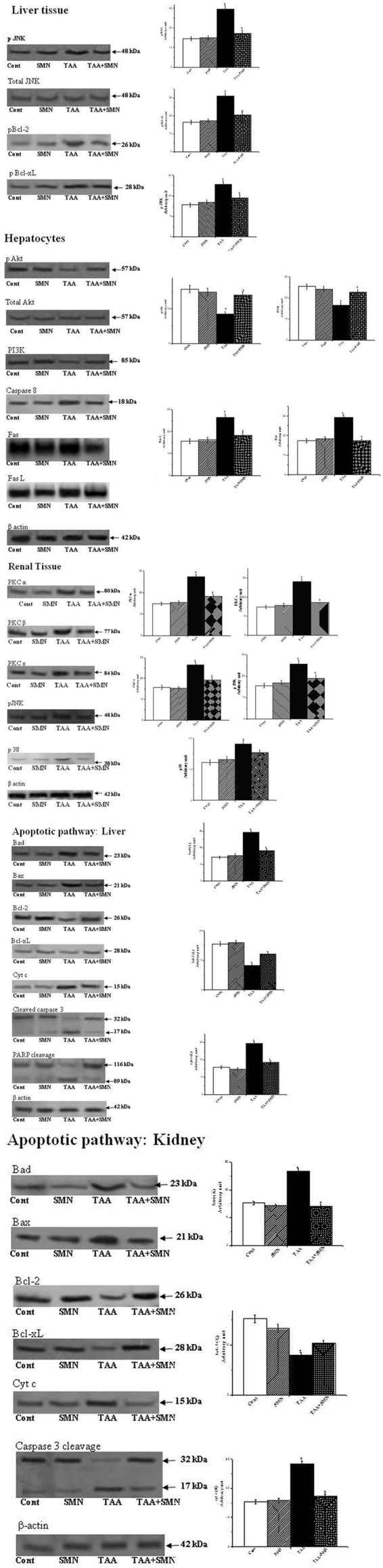
**Western blot analysis of different proteins.** β-actin served as a loading control. “a” indicates the significant difference between the normal and TAA-exposed animals liver and kidney tissue homogenates, “b” indicates the significant difference between TAA and SMN+TAA-treated animal tissue homogenates. Each column represents mean ± SEM, *n* = 6 (*p*^a^ < 0.05, *p*^b^ < 0.05).

The literature suggests that ROS, produced by the administration of various hepatotoxic agents, can activate JNK (Das et al., 2010; Du et al., 2014; Pal et al., 2015a). So, we first checked the level of JNK. Our result showed elevated level of JNK in TAA group. Two pathways may activate JNK. One of them involves oxidation of kinase inhibitors sequestering JNK or upstream molecules like ASK1. Alternatively, sustained JNK activation can be the result of inactivation of JNK phosphatases by ROS. Then, we wanted to check whether SMN could inhibit the phosphorylation and thereby activation of JNK as previous results showed that SMN treatment effectively inhibited JNK activation (Kim et al., 2016). JNK activation might be a potential regulator of mitochondrial permeabilization (Ghosh et al., 2010; Zhao et al., 2014). Phosphorylated JNK in turn phophorylates Bcl-2 family proteins such as Bcl-2 and Bcl-xL (Ma et al., 2014; Kim et al., 2015). Active Bcl-2 (unphosphorylated) maintains the balance between pro and anti-apoptotic proteins. So, once it is phosphorylated, that balance is lost and mitochondrial membrane depolarises resulting in pore formation starts ultimately leading to apoptosis. The result of our study showed that SMN post-treatment prevented the phosphorylation of Bcl-2 and resulting apoptosis (**Figure 6**).

### The Role of PI3K-Akt Cell Survival Pathway

The PI3K-Akt Pathway is a signaling pathway that regulates cell survival and growth in response to extracellular signals. Literature suggests that the PI 3-kinase/Akt signaling pathway plays an important role in cell survival in liver (Jiang et al., 2014; Rawat and Bouchard, 2015; Saha et al., 2016).

Recent report (Hawkins and Stephens, 2016) also suggest that class 1 PI3Ks, composed of a regulatory subunit p85 and a catalytic subunit p110, is stimulated by activated receptor tyrosine kinase (RTK). RTKs are activated by the binding of an extracellular ligand (SMN here) in the plasma membrane. p85 subunit then binds to phosphorylated tyrosine residues on the activated receptor via its SH2 domain and recruits the p110 forming the fully active PI3K enzyme. Activated PI3K phosphorylates phosphoinositides and forms phosphatidylinositol 3,4,5-triphosphate (PIP3) which facilitates the recruitment of Akt to the plasma membrane. Akt is phosphorylated by phosphoinositide-dependent kinase-1 (PDK1) at Thr308. Full activation of Akt, however, needs phosphorylation at Ser473 as well, by different other proteins like PDK-2, mTORC, etc., activated Akt then phosphorylates downstream molecules like BAD, caspase-9 and consequently initiates apoptosis. In this study, both the levels of PI 3-k and p-Akt levels were reduced in TAA-exposed murine liver compared with the control animals. These alterations in these protein levels due to TAA exposure were attenuated by treatment with SMN because of either increased synthesis or less degradation of these proteins in the presence of SMN (**Figure 6**).

### The Extrinsic Apoptotic Pathway in Hepatocytes

Literature reveals that high amounts of Fas, a member of the tumor necrosis factor (TNF) receptor superfamily (also known as CD95), are constitutively expressed on the surface of hepatocytes (Schneider et al., 1997; Wang et al., 2010). Fas plays a crucial role in case of many types of liver diseases also. After Fas ligand (FasL) binds to Fas on the cell surface, two apoptotic pathways can be initiated, a direct type I pathway and a mitochondria dependent type II pathway. Type I pathway starts with the formation of the death inducing signaling complex (DISK) at the activated Fas receptor. FasL binding initiates a conformational change. Consequently the intracellular domain of Fas is able to bind to the adaptor FADD which in turn activates caspase 8 in the cytosol from pro-caspase 8. Activated caspase 8 then proteolytically degrades pro-caspase 3 to form fully active caspase 3 which then initiates downstream signaling ultimately leading to apoptosis.

To know whether SMN could inhibit the Fas mediated (type I) apoptosis or not, we investigated the expression of capase 8 (at the protein level by western blot) and Fas and FasL (at the mRNA level by RT-PCR) (**Table 5**). Results showed that both the levels of Fas/FasL and caspase 8 have been suppressed significantly in the SMN post-treatment group, i.e., SMN protected TAA exposed mouse hepatocytes from Fas/FasL and caspase 8 mediated apoptosis effectively (**Figure 6**).

**Table 5:** The product size and annealing temperature of the primers used for Fas, Fas L, and actin genes.

Gene	Oligonucleotides used for real-time polymerase chain reaction (PCR) primer sequence 5′ to 3′	Annealing temperature (°C)	DNA bases (bp)
Fas	Fp:AGACAGGATGACCCTGAATCTA	54.7	22
	Bp:TTCTGCTCAGCTGTGTCTTG	55.1	20
Fas L	Fp:ATATGGGCCCACAGCAG	54.9	17
	Bp:AGACTCTCATTCAAGACAATATTCC	52.4	25
Actin	Fp:ACATTGGCATGGCTTTGTTT	53.8	20
	Bp:GTCCTCAGCCACATTTGTAGA	54.5	21

### PKC and MAPKs Mediated Renal Apoptosis

Next, we have searched about the involvement of ROS in renal dysfunction and the signaling pathways associated. Literature indicated that oxidative stress could induce PKC and MAPKs mediated renal tissue damage leading to apoptosis (Tuttle et al., 2005; Xu et al., 2010). So, we measured the above said signaling molecules quantitatively using western blot assay. The results of our study revealed that TAA exposure indeed increased the expression of different forms of PKC (PKCα, PKCβ, and PKC𝜀) and MAPKs (p38 and JNK). SMN treatment, however, restored their levels toward the normal value and protected the kidney (**Figure 6**).

### Apoptotic Cell Death Pathway both in Case of Liver and Kidney

Finally, we have investigated the intrinsic apoptotic cell death pathway in liver and renal tissues as TAA exposure might turn on this pathway. Apoptosis is one of the most complex fate of a cell primarily depends on the balance between pro-apoptotic and anti-apoptotic proteins. Apoptosis occurs when this balance is compromised either by the up-regulation of pro-apoptotic proteins and/or down regulation of anti-apoptotic proteins (Hui et al., 2016). The results of our study reveal that TAA increased the expressions of pro-apoptotic proteins, such as Bad and Bax and decreased the expressions of anti-apoptotic proteins Bcl-2 and Bcl-xL. Our results also showed that SMN post-treatment effectively ameliorated all of these anomalies.

In normal, healthy cells, pro-apoptotic protein Bax is present in the cytosol. When apoptotic signal is present, Bax undergoes a conformational change and translocated into the mitochondria and causes damages to the outer mitochondrial membrane causing pores on the mitochondrial outer membrane leading to mitochondrial membrane depolarisation (Gross et al., 1998; Bhattacharyya et al., 2014).

**FIGURE 7:**
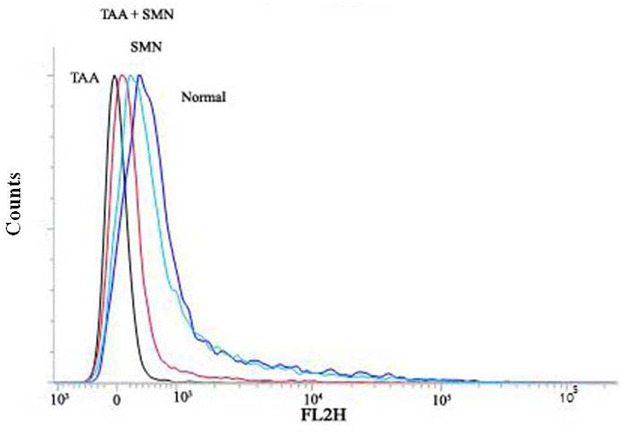
**Measurement of the mitochondrial membrane potential by flow cytometry analysis (using JC-1) from liver tissue homogenates**.

But maintenance of mitochondrial membrane potential (ψ_m_) is crucial for cellular survival as its (ψ_m_) loss induces the release of cytochrome c into the cytosol and activates downstream apoptotic signaling pathways (Sinha et al., 2013). Results of our present study showed that TAA exposure decreased the mitochondrial membrane potential (in hepatocytes) significantly and SMN could ameliorate this phenomenon effectively (**Figure 7**). Immunoblot studies also showed that cytosolic cytochrome c level was increased in TAA exposed group compared to normal. SMN post-treatment also protected mice liver and kidney from mitochondrial depolarisation directed cytochrome c release in the cytosol (**Figure 6**).

After the release into the cytosol, cytochrome c forms apoptosomes that activates caspase 9 and other downstream caspases (Eleftheriadis et al., 2016). In the present study, apoptosis was determined by the estimation of caspase-3 activation and PARP cleavage. Caspase 9 cleaves pro-caspase 3 and forms activated cleaved caspase 3, indicator of caspase-3 activation, which in turn, cleaves PARP and induces subsequent apoptosis (Pal et al., 2015b). Se, we measured the levels of cleaved caspase 3 and PARP. Both of these levels were significantly increased in TAA exposed liver and kidney tissues compared to normal animals. However, normalcy was restored in SMN post-treated group in both the cases. It is worth-mentioning that only SMN was not responsible for either caspase 3 activation or PARP-cleavage as evident from the results of SMN group (**Figure 6**).

## Conclusion

**FIGURE 8:**
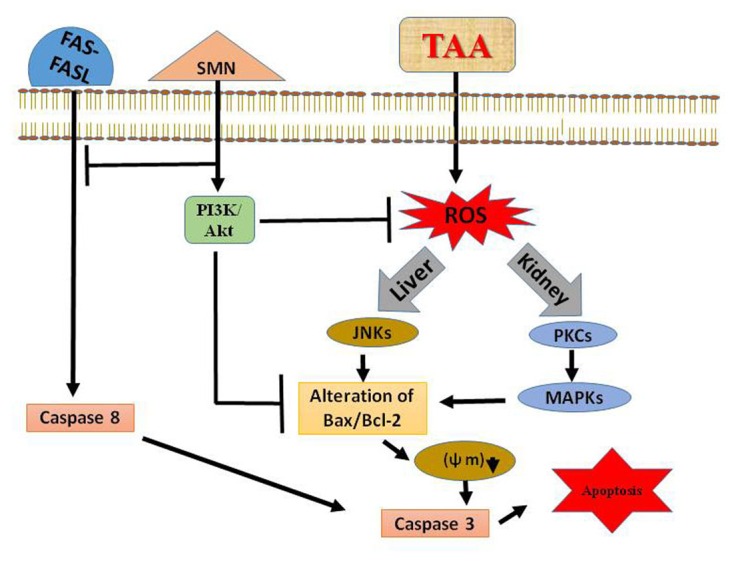
**Schematic diagram of the TAA induced hepatic as well as renal toxicity and its prevention by SMN**.

In summary, our study indicates that TAA generates oxidative stress that leads to systematic apoptosis within the liver (via the activation of JNK) and kidney (via PKC and MAPKs mediated molecular signaling pathways). SMN, a well-known antioxidant, might protect those organs by elevating the antioxidant enzyme activities and scavenging ROS as evidenced from biochemical results. Moreover SMN induces PI3K-Akt cell survival pathway in the liver and prevents apoptosis in both the organs (**Figure 8**). So, we are hopeful about the beneficial role of SMN in ameliorating liver as well as kidney under pathophysiologic conditions.

## Author Contributions

Experiment design: SG, AS, SB, and PS. Manuscript writing: SG, AS, SB, and PS. Results analysis: PS, SG, AS, and SB.

## Conflict of Interest Statement

The authors declare that the research was conducted in the absence of any commercial or financial relationships that could be construed as a potential conflict of interest.

## Abbreviations

ALP: alkaline phosphatase
ALT: alanine transaminase
AST: aspartate transaminase
CAT: catalase
GPx: glutathione peroxidase
GR: glutathione reductase
GST: glutathione *S*-transferase
LDH: lactate dehydrogenase
PARP: poly (ADP-ribose) polymerase 1
ROS: reactive oxygen species
SOD: superoxide dismutase
